# Rapid and complicated HIV genotype expansion among high-risk groups in Guangdong Province, China

**DOI:** 10.1186/s12879-019-3788-7

**Published:** 2019-02-22

**Authors:** Ping-Ping Zhou, Guolong Yu, Yi-Qun Kuang, Xu-He Huang, Yan Li, Xiaobing Fu, Peng Lin, Jin Yan, Xiang He

**Affiliations:** 1 Guangdong Provincial Institute of Public Health, Guangdong Provincial Center for Disease Control and Prevention, 160 Qunxian Road, Panyu District, Guangzhou, 511430 China; 2 Guangdong Provincial Center for Disease Control and Prevention, 160 Qunxian Road, Panyu District, Guangzhou, 511430 China; 3 Institute of Infection and Immunity, Henan University, Kaifeng, 475000 China

**Keywords:** HIV-1, Genotype, Risk factor, Molecular epidemiology, Guangdong, China

## Abstract

**Background:**

Guangdong Province is one of the most developed and populous provinces in southern China, with frequent foreign exchanges and large transient population. The annual number of cases of HIV/AIDS reported in Guangdong has been higher than most of provinces in China for several successive years. HIV infection by heterosexual transmission occurs across the province, with transmission among men who have sex with men occurring mainly in larger urban centers. There is a lack of widespread and representative data on the distribution of HIV subtypes in Guangdong. This study aimed to thoroughly investigate and estimate the prevalence and distribution of HIV-1 subtypes using a city-based sampling strategy to better understand the characteristics of HIV transmission in Guangdong.

**Methods:**

Archived plasma samples (*n* = 1205) from individuals diagnosed as HIV-1 infection in 2013 were selected randomly from all 21 cities in Guangdong Province. Genotypes were determined using *env* and/or *gag* sequences using phylogenetic analysis. The distributions of HIV genotypes in different risk groups and different cities were analyzed.

**Results:**

A total of 15 genotypes, including six discordant genotypes, were identified. The four main HIV-1 subtypes in Guangdong were CRF01_AE (43.2%), CRF07_BC (26.3%), CRF55_01B (8.5%), and CRF08_BC (8.4%). CRF01_AE was the predominant subtype in all risk populations. The high mobility of people shaped the complexity of the HIV genotypes, while the switch of risk factors affected the distribution and future trend of HIV-1 genotypes in Guangdong. Another epicenter located in the western region in addition to the known epicenter cities in the Pearl River Delta region of Guangdong may exist.

**Conclusions:**

Our study provides a comprehensive molecular epidemiologic dataset to understand the diversity and distribution of HIV genotypes in Guangdong, as well as to clarify the unique region- and risk group-specific transmission dynamics. The results provide critical and insightful information for more effective intervention strategies to limit HIV transmission in the future.

**Electronic supplementary material:**

The online version of this article (10.1186/s12879-019-3788-7) contains supplementary material, which is available to authorized users.

## Background

Guangdong Province (hereafter referred to as Guangdong) is located on the southern coast of China. It was one of the first provinces to establish relationships with the outside world, starting in 1978. The first imported case of human immunodeficiency virus (HIV) was identified in 1986 in Guangdong. Local HIV cases were reported in injection drug users (IDU) in 1996, with a subsequent rapid spread among drug users, with the number of cases reaching a peak in 2006 [1–3]. The sexual transmission of HIV began to increase in 2005 and by 2009 this route had become the predominant means of transmission, exceeding transmissions via IDU. It is noteworthy that HIV cases in men who have sex with men (MSM) accounted for over 10% of the infected population by 2013 [3]. The switch of major risk factors may have affected the distribution of HIV subtypes in Guangdong.

Before 2003, there were four prevalent HIV-1 genotypes in Guangdong—CRF01_AE, CRF07_BC, CRF08_BC, and subtype B—with CRF07_BC and CRF01_AE found mainly in IDU, CRF01_AE in cases of sexual transmission, and subtype B in blood donors and transfusion recipients [1, 4]. The examination of clinical samples in 2008 and 2009 studies revealed similar genotype distribution in Guangdong, with the exceptions of the newly detected subtype C, CRF02_AG, and discordant genotypes [5, 6]. Two studies in Guangdong focused on the HIV genotypes for different risk populations in the cities of as Guangzhou [7] and Shenzhen [8]. A recent publication described the genotype distribution and dynamics in HIV infected MSM in Shenzhen, and reported the complex HIV genotypes and emergence of new recombinants in this at-risk population [9]. However, the foregoing studies either involved limited sample sizes or focused on a specific location or risk population**,** and thus are not representative of the HIV genotype distribution across Guangdong.

Here we report a comprehensive study using a city-based sampling strategy to explore the geographic and demographic distribution of HIV genotypes across Guangdong. We estimated the likely prevalence and distribution of HIV-1 genotypes in Guangdong using the genotypic data of 1205 archived specimens combined with HIV case report data of various risk groups in prefecture-level cities in Guangdong. Our results provide a comprehensive dataset on the characteristics and diversity of HIV and acquired immunodeficiency syndrome (AIDS) in Guangdong.

## Methods

### Study participants and sampling strategy

A total of 1205 plasma specimens were randomly selected using the Random Sampling function in Excel software from HIV-1 positive samples previously collected and archived by Guangdong Provincial Center for Disease Control and Prevention, based on the reported case number of each city in Guangdong in 2013 (Table 1). The epidemiological data of the patients, including their risk group, gender, age, ethnicity, marital status, and education, were acquired from China Information System for Disease Control and Prevention. There were no significant differences in the risk groups between genotyped samples and reported cases for each city (all *p* > 0.05 using the χ^2^ test, Additional file 1: Table S1).

**Table 1:** HIV case reported in Guangdong in 2013 and sample selection

City	Number of reported cases	Number of selected samples	Sampling Ratio	Number of genotyped samples	Corrected number of represented cases in the study	Representation in the study
Guangzhou	1342	81	6.0%	77	1330	5.8%
Shenzhen	1189	80	6.7%	65	1187	5.5%
Dongguan	579	76	13.1%	67	575	11.7%
Foshan	482	76	15.8%	61	481	12.7%
Jiangmen	291	68	23.4%	60	291	20.6%
Yangjiang	187	90	48.1%	58	183	31.7%
Zhongshan	186	64	34.4%	60	186	32.3%
Zhanjiang	174	127	73.0%	55	174	31.6%
Huizhou	155	59	38.1%	59	154	38.3%
Yunfu	149	59	39.6%	45	147	30.6%
Maoming	147	59	40.1%	57	137	41.6%
Qingyuan	146	59	40.4%	59	143	41.3%
Zhaoqing	108	53	49.1%	50	108	46.3%
Shantou	91	50	54.9%	49	88	55.7%
Zhuhai	81	48	59.3%	48	81	59.3%
Shaoguan	61	31	50.8%	31	60	51.7%
Heyuan	48	33	68.8%	33	48	68.8%
Meizhou	47	30	63.8%	29	45	64.4%
Jieyang	40	20	50.0%	20	38	52.6%
Shanwei	24	20	83.3%	18	23	78.3%
Chaozhou	21	22	104.8%	20	21	95.2%
Total	5548	1205	21.7%	1021	5500	18.6%

### RNA extraction, amplification, and sequencing

Viral RNA was extracted from 200 μl of plasma using the MagPure Viral Nucleic Acid KF Kit (Magen, Guangzhou, China) with a KingFisher Flex Purification System (Thermo Fisher Scientific, Waltham, MA, USA) following the manufacturer’s instructions. Both *gag* (HXB2: 781–1836 nt) and *env* (HXB2: 7002–7645 nt) fragments were amplified using the primers and amplification conditions as previously described [10] with the PrimeScript One Step RT-PCR Kit and Premix Taq (Takara Bio, Dalian, China). PCR products were sent to a commercial company (Zixi Biotech, Beijing, China) for sequencing.

### Sequence analysis and subtype determination

The HIV sequences were aligned using the HIVAlign tool [6] (https://www.hiv.lanl.gov/content/sequence/VIRALIGN/viralign.html) and then merged with HIV-1 group M subtyping references from Los Alamos HIV Sequence Database using BioEdit [11]. HIV-1 genotypes were determined based on phylogenetic analysis. Neighbor-joining phylogenetic trees were reconstructed with the Kimura 2-parameter substitution model and evaluated by the bootstrap method with 1000 replicates using MEGA 5.2 [12]. The recombinants were screened by the RIP 3.0 tool [13].

The HIV-1 genotype of each patient was determined based on the genotypes of both *gag* and *env* fragments. If only one gene fragment was available, the genotype of that gene was assigned. Samples with different genotypes identified from *gag* and *env* regions were deemed discordant genotypes and were assigned *gag*/*env* genotype designations, such as CRF01/C.

### Estimation of HIV genotypes in different cities and risk groups

The HIV genotypes in different prefecture cities and risk groups were estimated according to previously described methods [6]. Briefly, the basic analysis unit in our study was a specific risk population in a city. The number of cases for each genotype in the unit was estimated by multiplying the proportion of that genotype in the unit with the total number of HIV cases reported in the unit. For a unit in which no specimen was obtained, the number of reported cases was excluded from the calculations. The adjusted totals and corresponding sampling ratios for each city are listed in the last two columns of Table 1. The estimated number of persons infected with each genotype in each risk group across the province was obtained by summing the units across all cities. Similarly, the estimated proportions and numbers of individuals infected with each genotype in each region and the province were obtained by summing up the units accordingly. The prevalence of HIV-1 genotypes was calculated by weighting the sampled prevalence with the size of the corresponding risk group in any given city; if no specimen was obtained for the risk- and city-specific subunit, the case count for that subunit was removed from the analysis. Approximately 0.9% (48 of 5548) reported cases were removed from the total case number (Table 1).

## Results

### Distribution of HIV-1 genotypes in Guangdong

A total of 1205 plasma specimens were selected from diagnosed HIV-positive persons, which constitute 21.7% of the total reported HIV-1 cases in 2013 in Guangdong. Among the samples, 1021 were determined for the HIV-1 genotypes from the 1.1-kb *gag* and/or the 563-bp *env* regions (Table 1). 862 *gag* genotypes (71.5% of samples) and 900 *env* genotypes (74.7%) were acquired, and both *gag* and *env* genotypes were assessable for 741 samples (61.5%).

**Fig. 1:**
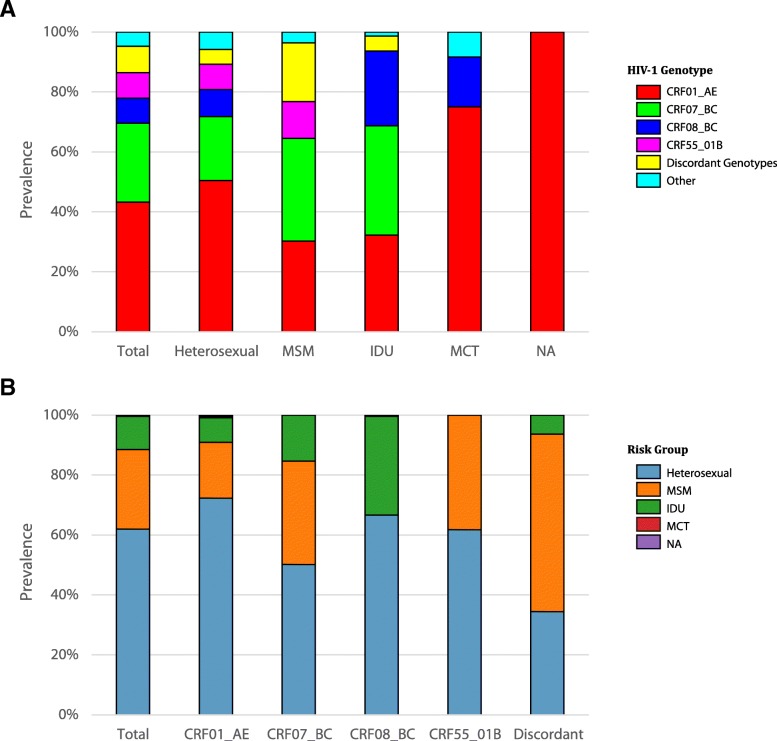
Proportion of HIV-1 genotypes in each risk group (**a**) and proportion of risk groups in each genotype category (**b**). The HIV-1 subtypes (CRF01_AE, CRF07_BC, CRF55_01B and CRF08_BC) were shown in colors among risk groups (X-axis). The bar chart depicts the percentage of subtypes and recombinant strains in respective risk groups (**a**). The risk groups (Heterosexual, MSM, IDU, MCT or NA) were shown in colors among HIV-1 subtypes (X-axis). The bar chart depicts the percentage of risk groups in respective genotypes (**b**)

Nine major HIV-1 subtypes and circulating recombinant forms (CRF) in total were detected in this study. They included subtypes B, B′ (Thai B strain), C, G, CRF01_AE, CRF07_BC, CRF08_BC, CRF55_01B, and CRF59_01B (Fig. 1a and Additional file 2: Table S2). CRF01_AE (43.2%), CRF07_BC (26.3%), CRF55_01B (8.5%), and CRF08_BC (8.4%) were the predominant HIV-1 subtypes circulating in Guangdong (Fig. 1a). These four subtypes were responsible for 86.4% of reported HIV infections in Guangdong in 2013. Minor HIV-1 genotypes included CRF59_01B (1.7%), B (1.1%), B′(0.7%), C (0.9%), and G (0.3%). They were classified as “other” genotypes. The remaining 8.8% were discordant genotypes between *gag* and *env* fragments, and included CRF01/C (4.1%), CRF07/CRF01 (3.1%), B/CRF01 (0.6%), CRF01/B (0.5%), 0107/CRF01 (0.4%), and BC/C (0.04%) (Additional file 2: Table S2).

### HIV-1 genotype distribution by risk group

As illustrated in Fig. 1b, sexual transmission including heterosexual and MSM was the primary risk factor in Guangdong, which accounted for 88.4% (61.9% for heterosexual and 26.5% for MSM) of HIV reported cases in 2013, followed by IDU including sexual plus IDU (11.1%) and mother to child transmission (MCT, 0.2%). About 0.2% of HIV cases resulted from unknown transmission routes.

CRF01_AE (50.5%), CRF07_BC (21.3%), CRF08_BC (9.0%), and CRF55_01B (8.5%) were the main HIV-1 subtypes circulating among heterosexuals, accounting for 89.3% of infections in this risk group, followed by discordant genotypes (4.9%) (Fig. 1a). The greatest genotype diversity was identified among heterosexuals, in which almost all the HIV-1 genotypes were detected, except B/CRF01. The main genotypes prevailing among MSM were CRF07_BC (34.2%), CRF01_AE (30.3%), and CRF55_01B (12.3%). Interestingly, CRF08_BC was not detected in MSM, while the discordant genotypes accounted for 19.6% of MSM infections, which was the highest ratio in all risk groups (Fig. 1a). Other genotypes in MSM were subtype B (1.8%), CRF59_01B (1.6%), and B′ (0.3%). Three main genotypes were detected in IDU, including CRF07_BC (36.5%), CRF01_AE (32.2%), and CRF08_BC (24.9%), followed by subtype C (1.3%) and discordant genotypes (5.1%). Only three HIV-1 genotypes, including CRF01_AE (75.0%), CRF08_BC (16.7%), and subtype C (8.3%), were detected among MCT.

### HIV-1 genotype distribution by geographic region

**Fig. 2:**
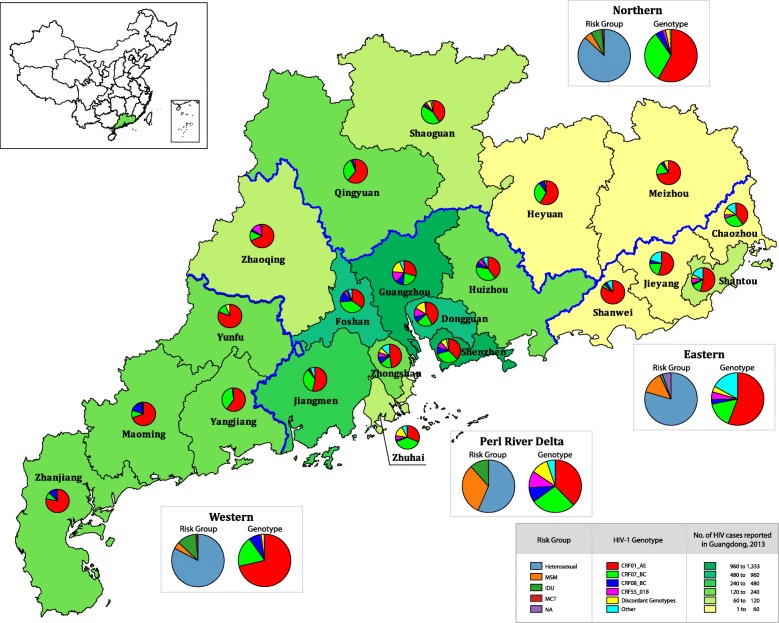
Distribution of HIV-1 genotypes in different cities of Guangdong. It is illustrated based on the dataset tabulated in Additional file 2: Table S2

According to the social-economic status, Guangdong could be classified into four regions: Pearl River Delta (PRD) region, eastern, western, and northern. The PRD region located in the central part of Guangdong has a prevalent Cantonese culture and is one of the most densely urbanized areas in the world, which includes the cities of Guangzhou, Shenzhen, Dongguan, Foshan, Jiangmen, Zhongshan, Huizhou, Zhaoqing, and Zhuhai. The eastern Guangdong is Teochew dialect-based and includes the four prefecture cities of Jieyang, Chaozhou, Shantou, and Shanwei. Northern Guangdong is dominated by mountains and includes four cities (Meizhou, Heyuan, Shaoguan, and Qingyuan). The Hakka dialects is prevalent in these cities. Western Guangdong is Cantonese culture based and includes the four cities of Zhanjiang, Maoming, Yangjiang, and Yunfu. As illustrated in Fig. 2, most HIV-1 cases in Guangdong were distributed in the PRD region (79.9%), followed by west (11.6%), north (5.4%), and east (3.1%) of Guangdong.

The estimated city-specific distributions of HIV-1 genotypes are presented in Fig. 2. In the PRD region, heterosexual (56.6%), MSM (31.8%), and IDU (11.6%) were the major risk groups. The PRD region comprised the highest genotype diversity, and almost all the genotypes, except BC/C, were identified in this region. CRF01_AE (37.7%), CRF07_BC (27.4%), CRF55_01B (10.3%), and discordant genotypes (10.4%) were the main genotype categories. Most of the cases of CRF55_01B (453 of 471, 96.2%) and discordant genotypes (459 of 485, 94.6%) were found in this region. Heterosexuals (60.9%) and MSM (39.1%) were the main risk groups for CRF55_01B, while MSM (62.3%) and heterosexuals (31.6%) were the main risk group for discordant genotypes.

In the western region, heterosexuals (82.7%) accounted for the majority of HIV-1 cases, followed by IDU (11.9%), MSM (4.2%), and MCT (1.3%). CRF01_AE (71.4%), CRF07_BC (18.8%), and CRF08_BC (7.0%) were the main prevailing HIV-1 strains, followed by CRF55_01B (0.6%), discordant genotypes (1.9%; 1.4% for CRF07/CRF01 and 0.5% for 0107/CRF01), and CRF59_01B (0.3%). Over 70% of HIV cases were infected with CRF01_AE in heterosexual (72.8%), MSM (74.1%), and MCT (75%) risk groups. The CRF01_AE composition ratio in the western region was the highest in all geographic regions, although the proportion of heterosexuals in the western region (82.7%) was less than that in the northern region (86.9%).

In the northern region, heterosexual (86.9%) was the predominant risk group, followed by IDU (6.7%), MSM (4.7%), and not available (NA, 1.3%). CRF01_AE (58.1%) and CRF07_BC (32.2%) were the main HIV-1 genotypes, followed by CRF08_BC (4.4%), CRF55_01B (2.0%), and discordant genotypes (2.7%; 1.3% for CRF07/CRF01, 1.0% for 0107/CRF01, and 0.3% for CRF01/C). Lower composition ratios for CRF01_AE were found in heterosexuals (60.6%), MSM (28.6%), and IDU (35%), while higher CRF07_BC ratios were found in those three risk groups.

In the eastern region, heterosexuals (79.4%) and MSM (13.5%) were the main risk groups, followed by IDU (1.8%) and NA (5.3%). CRF01_AE (55.9%), CRF07_BC (15.9%), and other genotypes (17.1%; 8.8% for subtype B, 5.3% for B′, and 2.9% for subtype C) were the main genotype categories in this region, followed by CRF55_01B (4.7%), CRF08_BC (2.9%), and discordant genotypes (3.5%; 1.2% for CRF07/CRF01, 1.2% for BC/C, 0.6% for CRF01/C, and 0.6% for 0107/CRF01). Among all geographic regions, the highest proportion of other genotypes category was observed in both heterosexuals (17.0%; 6.7% for subtype B, 6.7% for B′, and 3.7% for subtype C) and MSM (26.1%; all for subtype B) in the region. Of note, although the least number of HIV cases were reported in this region, there were 11 HIV-1 genotypes detected, which was the second highest number of genotypes.

### Distribution of HIV-1 strains in Guangdong by city and risk group

**Fig. 3:**
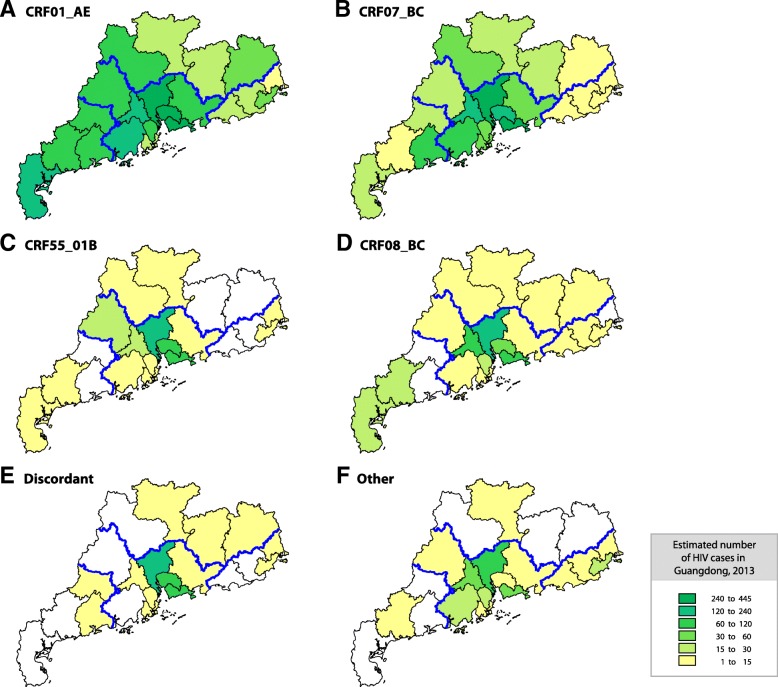
Estimated geographic distribution of the HIV-1 genotypes in Guangdong. The number of cases for the major HIV-1 genotypes, including CRF01_AE (**a**), CRF07_BC (**b**), CRF55_01B(**c**), CRF08_BC (**d**), Discordant genotypes (**e**) and Other genotypes (**f**), was estimated for each city. Corrected number of cases is color-coded as indicated in the inset

The geographic distributions of the main HIV-1 genotype categories in Guangdong are illustrated in Fig. 3. CRF01_AE was detected in all 21 cities accounting for 43.2% of reported HIV infections in Guangdong in 2013, which accounted for the highest proportion of HIV genotypes among 18 cities, except for Foshan, Zhuhai, and Shaoguan. The majority (69.7%) of CRF01_AE was distributed in the seven cities of Shenzhen (18.7%), Guangzhou (16.9%), Dongguan (10.1%), Foshan (7.0%), Jiangmen (6.5%), Zhanjiang (5.7%), and Yunfu (5.0%). The estimated number of CRF01_AE cases in Zhanjiang was 43.6% higher than that in the neighboring city of Maoming. It is conceivable that Zhanjiang was another possible epicenter for CRF01_AE in Guangdong along with Shenzhen and Guangzhou in the PRD region. The major risk groups for CRF01_AE were heterosexuals (72.3%) and MSM (18.6%), followed by IDU (8.3%) (Fig. 1b).

CRF07_BC was also detected in all cities in Guangdong. The majority (83.5%) of CRF07_BC infections were observed in the PRD region, especially in the five cities of Shenzhen (26.7%), Guangzhou (18.7%), Foshan (12.7%), Dongguan (8.5%), and Jiangmen (7.4%). Heterosexuals (50.1%), MSM (34.5%), and IDU (14.2%) were the main risk groups for CRF07_BC (Fig. 1b). Similar to CRF01_AE, the estimated number of CRF07_BC in Zhanjiang was higher than its nearby cities.

CRF55_01B was identified in 15 of 21 cities; the exceptions of were Yangjiang, Yunfu, Heyuan, Meizhou, Jieyang, and Shanwei. Most of the CRF55_01B cases were detected in the PRD region (96.2%) primarily in Guangzhou (42.9%), Shenzhen (22.5%), Dongguan (15.1%), and Foshan (5.5%). The two risk factors for CRF55_01B infections were heterosexual (61.8%) and MSM (38.2%).

CRF08_BC was detected in 18 cities (not in Yangjiang, Yunfu, and Chaozhou). The majority (83.5%) of CRF08_BC infections were identified in the PRD region (86.3%) and the western region (9.8%), primarily in Guangzhou (29.9%), Shenzhen (24.1%), Foshan (14.3%), Dongguan (10.0%), and Maoming (5.2%). Heterosexuals (66.6%) and IDUs (32.3%) were the primary risk populations for CRF08_BC infections (Fig. 1b), and no CRF08_BC was identified in MSM population.

Discordant genotypes were detected in 14 cities. Most (94.6%) were distributed in the PRD region, especially in Guangzhou (48.2%), Shenzhen (20.4%), and Dongguan (18.4%). MSM (59.2%) and heterosexuals (34.4%) were the main risk factors for the discordant genotypes in Guangdong, followed by IDU (6.4%).

CRF59_01B, subtypes B and B′, and subtypes C and G accounted for 35.5, 24.0, 15.3, 18.7 and 6.5% of the other genotypes, respectively. Most CRF59_01B infections were identified in the PRD region (97.8%) including Foshan (32.3%), Guangzhou (18.3%), Shenzhen (16.1%), Jiangmen (10.8%), Dongguan (9.7%), Zhongshan (4.3%), Zhuhai (4.3%), and Huizhou (2.2%), as well as the Maoming (2.2%) in the western region. CRF59_01B circulated in heterosexual (75.3%) and MSM (24.7%) groups. Subtypes B and B′ were identified in the PRD region (76.2 and 72.5%, respectively), the eastern region (23.8 and 22.5%, respectively), and the northern region (5.0% for B′). Heterosexuals comprised 58.7% of people with strain B and 90% of those with strain B′, whereas 41.3% of B and 10% of B′ were MSM. Subtype C was identified mainly in the PRD region (89.8%), including Guangzhou (34.7%), Jiangmen (20.4%), Zhongshan (20.4%), Huizhou (10.2%), and Zhaoqing (4.1%). There were also C infections in the eastern region, including Shantou (8.2%) and Chaozhou (2.0%). Heterosexual (81.6%), IDU (16.3%), and MCT (2.0%) were the risk factors for C infections. Subtype G was identified only in Guangzhou, and the risk factor was heterosexual transmission.

## Discussion

The predominant HIV-1 transmission route has changed with the changing socio-economic situations in China. Sexual transmission has been the main risk factor for HIV-1 infection, especially in the MSM group whose HIV-1 status remains unknown [14]. As one of regions on the forefront of the reform and opening of China, Guangdong has experienced similar trend and changes in HIV-1 risk factors [15]. We performed a comprehensive cross-sectional city-based survey in Guangdong using a molecular genotyping approach to understand the distribution characteristics of HIV genotypes. A total of 15 HIV-1 genotypes, including six discordant genotypes, were detected. This is more complicated than previously reported [6]. The HIV genotypic complexity in Guangdong may reflect the active mobility of people across the province.

The change of risk populations affected the distribution of HIV-1 genotypes and future trends in Guangdong. Similar to the HIV genotype survey in 2006 [6], the main prevailing HIV-1 genotypes in Guangdong remained CRF01_AE, CRF07_BC, and CRF08_BC. The proportion of CRF01_AE (including CRF55_01B and CRF59_01B) among HIV-1 infected people in Guangdong increased from 49.7% in 2006 to 53.5% in 2013 [6, 16, 17]. The proportion of CRF01_AE in heterosexually infected persons remained similar to 2006 (60.7%) and 2013 (61.0%), while the prevalence in IDU decreased from 44.1% in 2006 to 31.5% in 2013. However, the ratio of CRF01_AE in MSM unexpectedly increased from 33.3% in 2006 to 44.1% in 2013, which was also observed in nationwide studies [18, 19]. It is anticipated that the CRF01_AE infected cases will keep increasing if HIV infections in MSM continue growing rapidly. Similar to CRF01_AE, the proportion of CRF07_BC among HIV-1 infected people in Guangdong rose from 22.3% in 2006 to 26.3% in 2013. The percentage of CRF07_BC increased in IDU (from 23.5% in 2006 to 35.8% in 2013), heterosexuals (from 17.9 to 21.3% in the respective years), and MSM (from 33.3 to 34.2%) populations, which suggests that CRF07_BC would be the most likely genotype to increase in the near future. The proportion of HIV infected persons with CRF08_BC dropped drastically from 21.9% in 2006 to 8.4% in 2013. The percentage of CRF08_BC in IDU (including heterosexual plus IDU) dropped slightly from 29.4% in 2006 to 24.9% in 2013, and increased from 3.6% in 2006 to 9.0% in 2013 in heterosexual infected persons [6]. In Guangdong, the leading risk factor for HIV infection shifted from IDU (72.2%) in 2006 to heterosexual transmission (61.9%) in 2013, which is the primary cause for the decrease of the proportion of CRF08_BC. However, as the proportion of CRF08_BC in the heterosexual population increased over time, close attention should be paid to the future changes of the CRF08_BC [20]. In addition, it has been suggested that the CRF07_BC and CRF08_BC have been introduced into northern Myanmar via IDU, resulting in a subtype change related epidemic in Southeast Asia [21]. The extensive prevalence of subtypes like CRF01_AE, which was originally identified in Thailand and which has been transmitted across Thailand and other southeastern Asian countries predominantly through heterosexual behavior, is notable [22, 23].

As one of the most densely urbanized areas in the world and economically developed area in Guangdong, the PRD region has frequent foreign visitors and a sizable transient population. The high mobility of people has shaped the complexity of HIV genotypes in Guangdong. It is noteworthy that the proportion of HIV discordant genotypes was relatively high and increased from 0.6% in 2006 to 8.8% in 2013, which was much higher than that of national level in 2006 (2.6%) [6] or 2015 (3.8%, personal communication). Heterosexual sex (59.2%) has become the leading risk factor for the discordant genotypes, followed by MSM (34.4%) and IDU (6.4%), which is much different from that in 2006, when discordant genotypes were exclusively associated with MSM, and is consistent with other studies conducted in Guangdong [6, 9]. The increase of discordant genotypes circulating in the province suggests that there might be an active cross-transmission of HIV-1 genotypes among the high-risk populations. This provides further indication that the risk population switch has affected HIV genotype distribution. Additionally, a sentinel survey for MSM in Guangdong showed that 21.6 and 24% of MSM were married or had sex with women within the prior six months during the survey [24]. Therefore, considering the rapid transmission of HIV among MSM [18] and the sexual behaviors between women and MSM [25], along with the circulating CRF01_AE or the emergence of new second generation recombinants [26], the MSM group is still the great concern for HIV/AIDS interventions. More importantly, several studies also have observed the epidemic dynamics and complicated clusters of CRF01_AE in China [19, 27, 28]. With the generalization of the One Belt One Road Initiative, the communications between Asian and European countries are becoming more frequent. The burgeoning flow of people between China and global locales is increasing the challenge of infectious diseases [29], especially sexually transmitted diseases, such as HIV/AIDS, and for the introduction of new HIV recombinant forms [30].

Our updated data also reveal that Zhanjiang might be another potential epicenter in addition to Guangzhou and Shenzhen. In the city distribution pattern of HIV genotypes in Guangdong (Fig. 3), higher HIV-1 infected case numbers were found in Zhanjiang than the neighboring city of Maoming for both CRF01_AE and CRF07_BC. Especially, CRF01_AE in Zhanjiang was the highest in the west of Guangdong.

Our study has several limitations. Although the samples and the genotypes in our study accounted for 21.9 and 18.4% of all HIV infections in Guangdong in 2013, respectively, there may have been sampling bias due to the limited sample number for some risk populations and cities, for instance, IDU in Guangzhou or Yunfu. Also, since the positive ratio for PCR amplification with samples in 2013 from Chaozhou and Shanwei was low, we selected some archived samples from patients reported in 2014 as a supplement to those two cities.

In summary, our study clarifies the recent state of the HIV/AIDS epidemic in Guangdong. The newly-revealed regional and risk group-specific transmission patterns provide information that will be critical for developing effective interventions against HIV transmission in Guangdong.

## Conclusions

Our study provides a comprehensive molecular epidemiologic dataset to understand the diversity and distribution of HIV genotypes in Guangdong, as well as to clarify the unique region- and risk group-specific transmission dynamics. The results provide critical and insightful information for more effective intervention strategies to limit HIV transmission in the future.

## Additional files

Additional file 1: **Table S1** Representative evaluation of genotyped samples by city in the study. (PDF 87 kb)

Additional file 2: **Table S2** Estimated number of HIV-1 genotype in different cities and risk groups. (PDF 215 kb)

## Abbreviations

AIDS: Acquired immunodeficiency syndrome
CRF: Circulating recombinant form
HIV: Human immunodeficiency virus
IDU: Injecting drug user
MCT: Mother to child transmission
MSM: Men who have sex with men
NA: Not available
OBOR: One Belt One Road Initiative
PRD region: Pearl River Delta region
χ^2^: Chi squared test
