# Supplementary material for: Rapid and complicated HIV genotype expansion among high-risk groups in Guangdong Province, China
Source: BMC Infect Dis. 2019 Feb 22;19:185. doi: 10.1186/s12879-019-3788-7 (PMC6387515; doi:10.1186/s12879-019-3788-7)
Supplement: Supplementary file 1 — Table S1 Representative evaluation of genotyped samples by city in the study. (PDF 87 kb) [file 12879_2019_3788_MOESM1_ESM.pdf]

Table S1. Representative evaluation of genotyped samples by city in the study.

Region	City Risk Group	Reported cases	Genotyped samples	Chi-square	*P* value
PRD	**Guangzhou**				
	IDU	110	4	1.673	0.643
	Sex+IDU	7	1		
	Heterosexual	715	43		
	MSM	498	29		
	**Shenzheng**				
	IDU	88	5	4.569	0.206
	Sex+IDU	19	2		
	Heterosexual	533	21		
	MSM	547	37		
	**Dongguan**				
	IDU	40	6	1.303	0.728
	Sex+IDU	3	1		
	Heterosexual	392	44		
	MSM	140	16		
	**Foshan**				
	IDU	102	14	1.002	0.606
	Heterosexual	291	39		
	MSM	88	8		
	**Jiangmen**				
	IDU	72	15	0.172	0.982
	Sex+IDU	3	1		
	Heterosexual	196	40		
	MSM	20	4		
	**Zhongshan**				
	IDU	18	7	1.496	0.683
	Heterosexual	123	41		
	MSM	44	11		
	MCT	1	1		
	**Huizhou**				
	IDU	23	10	0.192	0.979
	Heterosexual	111	41		
	Sex+IDU	3	1		
	MSM	17	7		
	**Zhaoqing**				
	IDU	12	9	1.826	0.609
	Heterosexual	92	39		
	MSM	3	1		
	MCT	1	1		
	**Zhuhai**				
	IDU	5	2	0.550	0.760
	Heterosexual	37	20		
	MSM	39	26		
Western	**Yangjiang**				
	IDU	57	19	2.421	0.298
	Heterosexual	123	36		
	MCT	3	3		
	**Zhanjiang**				
	IDU	12	3	3.683	0.298
	Heterosexual	140	40		
	MSM	19	9		
	MCT	3	3		
	**Yunfu**				
	IDU	7	1	0.559	0.756
	Heterosexual	137	43		
	MSM	3	1		
	**Maoming**				
	Heterosexual	130	54	0.025	0.988
	MSM	5	2		
	MCT	2	1		
Northern	**Qingyuan**				
	IDU	5	4	1.971	0.373
	Heterosexual	136	53		
	MSM	2	2		
	**Shaoguan**				
	IDU	6	3	0.526	0.769
	Heterosexual	45	25		
	MSM	9	3		
	**Heyuan**				
	IDU	9	5	0.844	0.839
	Heterosexual	34	25		
	MSM	2	2		
	NA	3	1		
	**Meizhou**				
	Heterosexual	42	27	0.141	0.932
	MSM	1	1		
	MCT	2	1		
Eastern	**Shantou**				
	Heterosexual	74	40	0.136	0.712
	MSM	14	9		
	**Jieyang**				
	Heterosexual	35	18	0.074	0.786
	MSM	3	2		
	**Shanwei**				
	IDU	3	3	2.475	0.480
	Heterosexual	12	11		
	MSM	1	1		
	NA	9	3		
	**Chaozhou**				
	Heterosexual	17	16	0.006	0.939
	MSM	4	4		
