# Supplementary material for: Rapid and complicated HIV genotype expansion among high-risk groups in Guangdong Province, China
Source: BMC Infect Dis. 2019 Feb 22;19:185. doi: 10.1186/s12879-019-3788-7 (PMC6387515; doi:10.1186/s12879-019-3788-7)
Supplement: Supplementary file 2 — Table S2 Estimated number of HIV-1 genotype in different cities and risk groups. (PDF 215 kb) [file 12879_2019_3788_MOESM2_ESM.pdf]

Table S2. Estimated number of HIV-1 genotype in different cities and risk groups.

Region	City	Risk group	01_AE	07_BC	08_BC	55_01B	Other*	DG*	Total	B	B'	59_01B	C	G	01C	0701	B01	01B	0107	BC
PRD	**Guangzhou**		**402**	**272**	**138**	**202**	**85**	**234**	**1333**	**17**	**17**	**17**	**17**	**17**	**97**	**86**	**17**	**17**	**17**	**0**
		IDU	0	28	55	0	0	28	111	0	0	0	0	0	28	0	0	0	0	0
		Sex+IDU	0	7	0	0	0	0	7	0	0	0	0	0	0	0	0	0	0	0
		Heteosexual	299	100	83	116	68	51	717	0	17	17	17	17	17	17	0	17	0	0
		MSM	103	137	0	86	17	155	498	17	0	0	0	0	52	69	17	0	17	0
	**Shenzhen**		**445**	**387**	**111**	**106**	**40**	**99**	**1188**	**25**	**0**	**15**	**0**	**0**	**69**	**15**	**15**	**0**	**0**	**0**
		IDU	35	18	35	0	0	0	88	0	0	0	0	0	0	0	0	0	0	0
		Sex+IDU	10	10	0	0	0	0	20	0	0	0	0	0	0	0	0	0	0	0
		Heteosexual	178	152	76	76	25	25	532	25	0	0	0	0	25	0	0	0	0	0
		MSM	222	207	0	30	15	74	548	0	0	15	0	0	44	15	15	0	0	0
	**Dongguan**		**240**	**123**	**46**	**71**	**9**	**89**	**578**	**0**	**0**	**9**	**0**	**0**	**44**	**45**	**0**	**0**	**0**	**0**
		IDU	27	7	7	0	0	0	41	0	0	0	0	0	0	0	0	0	0	0
		Sex+IDU	0	0	3	0	0	0	3	0	0	0	0	0	0	0	0	0	0	0
		Heteosexual	169	98	36	36	9	45	393	0	0	9	0	0	9	36	0	0	0	0
		MSM	44	18	0	35	0	44	141	0	0	0	0	0	35	9	0	0	0	0
	**Foshan**		**168**	**184**	**66**	**26**	**30**	**7**	**481**	**0**	**0**	**30**	**0**	**0**	**0**	**0**	**0**	**7**	**0**	**0**
		IDU	15	51	36	0	0	0	102	0	0	0	0	0	0	0	0	0	0	0
		Heteosexual	142	67	30	15	30	7	291	0	0	30	0	0	0	0	0	7	0	0
		MSM	11	66	0	11	0	0	88	0	0	0	0	0	0	0	0	0	0	0
	**Jiangmen**		**154**	**108**	**5**	**5**	**20**	**0**	**292**	**0**	**0**	**10**	**10**	**0**	**0**	**0**	**0**	**0**	**0**	**0**
		IDU	26	41	0	0	5	0	72	0	0	0	5	0	0	0	0	0	0	0
		Sex+IDU	3	0	0	0	0	0	3	0	0	0	0	0	0	0	0	0	0	0
		Heteosexual	115	57	5	5	15	0	197	0	0	10	5	0	0	0	0	0	0	0
		MSM	10	10	0	0	0	0	20	0	0	0	0	0	0	0	0	0	0	0
	**Zhongshan**		**89**	**30**	**17**	**13**	**24**	**14**	**187**	**3**	**7**	**4**	**10**	**0**	**11**	**3**	**0**	**0**	**0**	**0**
		IDU	5	3	8	0	3	0	19	0	0	0	3	0	0	0	0	0	0	0
		Heteosexual	72	15	9	9	12	6	123	3	3	0	6	0	3	3	0	0	0	0
		MSM	12	12	0	4	8	8	44	0	4	4	0	0	8	0	0	0	0	0
		MCT	0	0	0	0	1	0	1	0	0	0	1	0	0	0	0	0	0	0
	**Huizhou**		**61**	**58**	**10**	**10**	**12**	**3**	**154**	**0**	**5**	**2**	**5**	**0**	**0**	**3**	**0**	**0**	**0**	**0**
		IDU	7	12	5	0	0	0	24	0	0	0	0	0	0	0	0	0	0	0
		Sex+IDU	3	0	0	0	0	0	3	0	0	0	0	0	0	0	0	0	0	0
		Heteosexual	46	41	5	5	10	3	110	0	5	0	5	0	0	3	0	0	0	0
		MSM	5	5	0	5	2	0	17	0	0	2	0	0	0	0	0	0	0	0
	**Zhaoqing**		**74**	**15**	**3**	**15**	**2**	**0**	**109**	**0**	**0**	**0**	**2**	**0**	**0**	**0**	**0**	**0**	**0**	**0**
		IDU	7	3	3	0	0	0	13	0	0	0	0	0	0	0	0	0	0	0
		Heteosexual	66	12	0	12	2	0	92	0	0	0	2	0	0	0	0	0	0	0
		MSM	0	0	0	3	0	0	3	0	0	0	0	0	0	0	0	0	0	0
		MCT	1	0	0	0	0	0	1	0	0	0	0	0	0	0	0	0	0	0
	**Zhuhai**		**27**	**32**	**2**	**5**	**7**	**13**	**86**	**3**	**0**	**4**	**0**	**0**	**5**	**4**	**0**	**4**	**0**	**0**
		IDU	3	3	0	0	0	0	6	0	0	0	0	0	0	0	0	0	0	0
		Heteosexual	19	6	2	2	2	8	39	0	0	2	0	0	2	4	0	2	0	0
		MSM	5	23	0	3	5	5	41	3	0	2	0	0	3	0	0	2	0	0
Western	**Yangjiang**		**109**	**71**	**0**	**0**	**0**	**3**	**183**	**0**	**0**	**0**	**0**	**0**	**0**	**3**	**0**	**0**	**0**	**0**
		IDU	27	27	0	0	0	3	57	0	0	0	0	0	0	3	0	0	0	0
		Heteosexual	79	44	0	0	0	0	123	0	0	0	0	0	0	0	0	0	0	0
		MCT	3	0	0	0	0	0	3	0	0	0	0	0	0	0	0	0	0	0

	**Zhanjiang**	**135**	**16**	**21**	**2**	**0**	**0**	**174**	**0**	**0**	**0**	**0**	**0**	**0**	**0**	**0**	**0**	**0**	**0**
	IDU	12	0	0	0	0	0	12	0	0	0	0	0	0	0	0	0	0	0
	Heteosexual	105	14	21	0	0	0	140	0	0	0	0	0	0	0	0	0	0	0
	MSM	15	2	0	2	0	0	19	0	0	0	0	0	0	0	0	0	0	0
	MCT	3	0	0	0	0	0	3	0	0	0	0	0	0	0	0	0	0	0
	**Yunfu**	**119**	**19**	**0**	**0**	**0**	**9**	**147**	**0**	**0**	**0**	**0**	**0**	**0**	**6**	**0**	**0**	**3**	**0**
	IDU	7	0	0	0	0	0	7	0	0	0	0	0	0	0	0	0	0	0
	Heteosexual	112	16	0	0	0	9	137	0	0	0	0	0	0	6	0	0	3	0
	MSM	0	3	0	0	0	0	3	0	0	0	0	0	0	0	0	0	0	0
	**Maoming**	**94**	**14**	**24**	**2**	**2**	**0**	**136**	**0**	**0**	**2**	**0**	**0**	**0**	**0**	**0**	**0**	**0**	**0**
	Heteosexual	89	14	22	2	2	0	129	0	0	2	0	0	0	0	0	0	0	0
	MSM	5	0	0	0	0	0	5	0	0	0	0	0	0	0	0	0	0	0
	MCT	0	0	2	0	0	0	2	0	0	0	0	0	0	0	0	0	0	0
**Northern**	**Qingyuan**	**87**	**47**	**5**	**4**	**0**	**0**	**143**	**0**	**0**	**0**	**0**	**0**	**0**	**0**	**0**	**0**	**0**	**0**
	IDU	0	5	0	0	0	0	5	0	0	0	0	0	0	0	0	0	0	0
	Heteosexual	87	41	5	3	0	0	136	0	0	0	0	0	0	0	0	0	0	0
	MSM	0	1	0	1	0	0	2	0	0	0	0	0	0	0	0	0	0	0
	**Shaoguan**	**25**	**26**	**2**	**2**	**2**	**4**	**61**	**0**	**2**	**0**	**0**	**0**	**0**	**4**	**0**	**0**	**0**	**0**
	IDU	0	6	0	0	0	0	6	0	0	0	0	0	0	0	0	0	0	0
	Heteosexual	22	14	2	2	2	4	46	0	2	0	0	0	0	4	0	0	0	0
	MSM	3	6	0	0	0	0	9	0	0	0	0	0	0	0	0	0	0	0
	**Heyuan**	**28**	**15**	**4**	**0**	**0**	**1**	**48**	**0**	**0**	**0**	**0**	**0**	**1**	**0**	**0**	**0**	**0**	**0**
	IDU	7	2	0	0	0	0	9	0	0	0	0	0	0	0	0	0	0	0
	Heteosexual	18	11	4	0	0	1	34	0	0	0	0	0	1	0	0	0	0	0
	MSM	0	2	0	0	0	0	2	0	0	0	0	0	0	0	0	0	0	0
	NA	3	0	0	0	0	0	3	0	0	0	0	0	0	0	0	0	0	0
	**Meizhou**	**33**	**8**	**2**	**0**	**0**	**3**	**46**	**0**	**0**	**0**	**0**	**0**	**0**	**0**	**0**	**0**	**3**	**0**
	Heteosexual	30	8	2	0	0	3	43	0	0	0	0	0	0	0	0	0	3	0
	MSM	1	0	0	0	0	0	1	0	0	0	0	0	0	0	0	0	0	0
	MCT	2	0	0	0	0	0	2	0	0	0	0	0	0	0	0	0	0	0
**Eastern**	**Shantou**	**47**	**12**	**2**	**7**	**16**	**4**	**88**	**10**	**2**	**0**	**4**	**0**	**0**	**2**	**0**	**0**	**0**	**2**
	Heteosexual	44	7	2	7	10	4	74	4	2	0	4	0	0	2	0	0	0	2
	MSM	3	5	0	0	6	0	14	6	0	0	0	0	0	0	0	0	0	0
	**Jieyang**	**21**	**8**	**2**	**0**	**8**	**0**	**39**	**4**	**4**	**0**	**0**	**0**	**0**	**0**	**0**	**0**	**0**	**0**
	Heteosexual	19	6	2	0	8	0	35	4	4	0	0	0	0	0	0	0	0	0
	MSM	2	2	0	0	0	0	4	0	0	0	0	0	0	0	0	0	0	0
	**Shanwei**	**19**	**1**	**1**	**0**	**2**	**0**	**23**	**0**	**2**	**0**	**0**	**0**	**0**	**0**	**0**	**0**	**0**	**0**
	IDU	3	0	0	0	0	0	3	0	0	0	0	0	0	0	0	0	0	0
	Heteosexual	7	0	1	0	2	0	10	0	2	0	0	0	0	0	0	0	0	0
	MSM	0	1	0	0	0	0	1	0	0	0	0	0	0	0	0	0	0	0
	NA	9	0	0	0	0	0	9	0	0	0	0	0	0	0	0	0	0	0
	**Chaozhou**	**8**	**6**	**0**	**1**	**3**	**2**	**20**	**1**	**1**	**0**	**1**	**0**	**1**	**0**	**0**	**0**	**1**	**0**
	Heteosexual	6	5	0	1	3	1	16	1	1	0	1	0	1	0	0	0	0	0
	MSM	2	1	0	0	0	1	4	0	0	0	0	0	0	0	0	0	1	0
	**Total**	**2385**	**1452**	**461**	**471**	**262**	**485**	**5516**	**63**	**40**	**93**	**49**	**17**	**228**	**171**	**32**	**28**	**24**	**2**

* Other includes subtype B, B', CRF59_01B, C and G; DG represents discordant genotypes including CRF01/C, CRF07/CRF01, B/CRF01, CRF01/B, 0107/CRF01, and BC/C.
